# F23. LOW-GRADE INFLAMMATORY PROFILE IN FIRST-EPISODE PSYCHOSIS: RESULTS FROM THE STREAM STUDY IN BRAZIL

**DOI:** 10.1093/schbul/sby017.554

**Published:** 2018-04-01

**Authors:** Fabiana Corsi-Zuelli, Camila Loureiro, Fachim Helene Aparecida, Rosana Shuhama, Menezes Paulo Rossi, Paulo Louzada-Junior, Del-Ben Cristina Marta

**Affiliations:** University of São Paulo, Ribeirão Preto School of Medicine

## Abstract

**Background:**

The inflammatory hypothesis of schizophrenia suggests that abnormalities in inflammatory factors may contribute to the onset of this disease early in adulthood. Nevertheless, no study has investigated possible effects of shared environment by looking at cytokine levels in healthy siblings of first-episode psychosis patients (FEP). The aim of this study was to investigate inflammatory cytokine (IL-6, IL-10, TNF-a) abnormalities in a sizeable epidemiological-based sample of FEP patients, their healthy siblings and population-based controls.

**Methods:**

This study is part of the epidemiological investigation “Schizophrenia and Other Psychoses Translational Research: Environment and Molecular Biology” (STREAM), conducted in Ribeirão Preto (São Paulo, Brazil) and surrounding area, which is part of the international consortium “European Network of National Schizophrenia Networks Studying Gene-Environment Interactions” (EU-GEI). We recruited a total sample of 507 participants, composed by 166 FEP patients (64% males; mean age: 30.3 ± 12.2), 76 siblings (30.3% males; mean age: 31.5 ± 11.0) and 265 population-based controls (50.2% males; mean age: 31.7 ± 11.2). Plasma cytokines IL-6, TNF-a and IL-10 were analysed by Multiplex Bead Array (Luminex xMap technology). We performed the multivariate general linear model (GLM) analysis with age as covariate, cytokines as dependent variables and diagnostic group and sex as explanatory variables.

**Results:**

We found significant differences between the three groups [F (6,994) = 10.864; p < 0.001] in the plasma concentration of all the three cytokines (p < 0.001), independent of the sex of the participants (p = 0.115; interaction diagnosis & sex p = 0.115). FEP patients had significantly higher levels of IL-6, TNF-a, and IL-10 when compared to controls (p < 0.001 for all). When compared to their siblings, patients had higher levels of both TNF-a and IL-10 (p < 0.05 for both), and a trend to IL-6 (p = 0.058), whereas siblings did not differ from controls for any of the three cytokines analysed (p > 0.05 for all).

**Discussion:**

This is the first study conducted in the South hemisphere to demonstrate the low-grade inflammatory profile in FEP patients, compared to their siblings and community-based controls. The fact that IL-6, TNF-a and IL-10 are all higher in the FEP group than their healthy siblings and community-based controls suggests the synergism of individual vulnerability factors in the development of this inflammatory profile.

